# SPIN90 dephosphorylation is required for cofilin-mediated actin depolymerization in NMDA-stimulated hippocampal neurons

**DOI:** 10.1007/s00018-013-1391-4

**Published:** 2013-06-14

**Authors:** In Ha Cho, Min Jung Lee, Dae Hwan Kim, Bora Kim, Jeomil Bae, Kyu Yeong Choi, Seon-Myung Kim, Yun Hyun Huh, Kun Ho Lee, Chong-Hyun Kim, Woo Keun Song

**Affiliations:** 1 Bio Imaging and Cell Dynamics Research Center, Gwangju Institute of Science and Technology, 261 Cheomdan-gwagiro, Buk-Gu, Gwangju, 500-712 Korea; 2 Department of Neurology, Program of Developmental Neurobiology, Institute of Molecular Medicine and Genetics, Medical College of Georgia, Augusta, 30912 GA USA; 3 Department of Marine Biology, Chosun University, Gwangju, 501-759 Korea; 4 Seoul and Department of Neuroscience, Center for Neural Science, Korea Institute of Science and Technology, University of Science and Technology, Daejeon, Korea

**Keywords:** Dendritic spines, Long-term depression, Spine shrinkage, Actin depolymerization

## Abstract

Actin plays a fundamental role in the regulation of spine morphology (both shrinkage and enlargement) upon synaptic activation. In particular, actin depolymerization is crucial for the spine shrinkage in NMDAR-mediated synaptic depression. Here, we define the role of SPIN90 phosphorylation/dephosphorylation in regulating actin depolymerization via modulation of cofilin activity. When neurons were treated with NMDA, SPIN90 was dephosphorylated by STEP61 (striatal-enriched protein tyrosine phosphatase) and translocated from the spines to the dendritic shafts. In addition, phosphorylated SPIN90 bound cofilin and then inhibited cofilin activity, suggesting that SPIN90 dephosphorylation is a prerequisite step for releasing cofilin so that cofilin can adequately sever actin filaments into monomeric form. We found that SPIN90 YE, a phosphomimetic mutant, remained in the spines after NMDAR activation where it bound cofilin, thereby effectively preventing actin depolymerization. This led to inhibition of the activity-dependent redistribution of cortactin and drebrin A, as well as of the morphological changes in the spines that underlie synaptic plasticity. These findings indicate that NMDA-induced SPIN90 dephosphorylation and translocation initiates cofilin-mediated actin dynamics and spine shrinkage within dendritic spines, thereby modulating synaptic activity.

**Electronic supplementary material:**

The online version of this article (doi:10.1007/s00018-013-1391-4) contains supplementary material, which is available to authorized users.

## Introduction

Actin is a major cytoskeletal protein found in dendritic spines, and actin dynamics play a central role in the regulation of spine morphogenesis, which is in turn closely associated with synaptic plasticity. Indeed, it has been proposed that actin dynamics regulate long-term plasticity [1]. It is therefore not surprising that many proteins regulating actin dynamics affect synaptic plasticity. For example, PICK1 regulates spine shrinkage during LTD by inhibiting Arp2/3 activity [2], while kalirin-7, a Rho guanine nucleotide exchange factor localized in spines, modulates dendritic spine morphology by activating Rac and induces spine enlargement during long-term potentiation (LTP) [3]. Another particularly interesting example is cofilin, which is a key regulator of actin dynamics and is essential for synaptic plasticity. Not only does cofilin reportedly trigger the spine shrinkage and loss associated with LTD, it also mediates the spine enlargement and AMPAR trafficking associated with LTP [4, 5]. However, it still remains obscure how cofilin activity is regulated during synaptic stimulation.

Postsynaptic densities (PSDs) located at the tip of dendritic spines are microscopic structures composed of glutamate receptors, scaffold and cytoskeletal proteins, and signal transduction molecules [6]. PSDs undergo rapid and remarkable remodeling of their structure and function in response to synaptic stimuli [7]. In particular, actin-related proteins, which are well organized in dendritic spines, undergo dramatic changes in their localization that underlie synaptic plasticity. For example, synaptic activity leads to a redistribution of β-catenin to the spines, which enables increased association between β-catenin and cadherins and increases the size and density of PSDs [8]. The sequestering of cortactin away from the spines in response to NMDAR activation may deactivate Arp2/3-mediated actin polymerization and thus lead to reorganization of the actin in the spines [9]. Drebrin A, another F-actin binding protein, is localized in the dendritic spines and translocates to the dendritic shaft on NMDAR activation [10]. Alteration of the distribution of drebrin A in neurons is thought to be important for the actin dynamics that accompany synaptic plasticity [11]. Therefore, abnormal distribution or expression of various PSD proteins in neurons adversely affects synaptic plasticity.

SPIN90 was originally identified as a binding partner of Nck [12]. It is strongly expressed in many tissues, especially heart, testis, and brain. The previous studies revealed that SPIN90 acts on the actin cytoskeleton, playing a key role during several actin-related processes, such as Rac-induced membrane ruffling and sarcomere assembly [12–14]. We also showed previously that SPIN90 is located in brain regions where F-actin is enriched [15, 16] and that genetic disruption of SPIN90 caused a dramatic reduction of F-actin in dendritic spines, suggesting that SPIN90 participates in the actin dynamics that contribute to the regulation of spine morphology [17]. However, the role of SPIN90 in response to synaptic stimulation is unclear.

In this study, we show that dephosphorylation of SPIN90 by STEP61 in response to NMDAR activation leads to its redistribution from the dendritic spine to the shaft. Moreover, a phosphomimetic mutant of SPIN90 remained in the spines and prevented cofilin-mediated actin depolymerization and translocation of drebrin A and cortactin. These findings indicate that phosphorylation/dephosphorylation of SPIN90 alters its localization in spines, and affects the translocation of other actin-related proteins, thereby regulating spine shrinkage.

## Materials and methods

### Antibodies and reagents

Mouse monoclonal antibodies were used against PSD95, drebrin (Abcam), phosphotyrosine, phosphoserine, HA, cortactin, GluA1 (Millipore, Bedford, MA, USA), GFP, phosphothreonine (Invitrogen, Carlsbad, CA, USA), α-tubulin, Flag (Sigma) and STEP (Cell Signaling Technology, Beverly, MA, USA). Mouse monoclonal anti-Myc (clone 9E10) antibody was generated from mouse ascites after hybridoma injection. Rabbit polyclonal antibodies were used against Vamp2 (ABR), cortactin, cofilin (Santa Cruz Biotechnology, Santa Cruz, CA, USA), pS845 GluA1 (Millipore), Slingshot-1L (ECM Biosciences, Versailles, KY, USA), and pY416-Src (Cell Signaling Technology). Rabbit polyclonal anti-SPIN90 serum was described in previous studies [15], and rabbit polyclonal anti-Shank antibody was gifted by Dr. Eunjoon Kim (KAIST, Republic of Korea). Goat polyclonal antibody (Santa Cruz) was used against actin. Horseradish peroxidase-conjugated anti-mouse, anti-rabbit and anti-goat secondary antibodies were purchased from the Jackson Laboratory (Bar Harbor, MA, USA). Alexa Fluor 488-, 555-, 594-, and 647-conjugated donkey anti-rabbit IgG, donkey anti-mouse IgG were from Molecular Probes. NMDA was purchased from Calbiochem, APV (D-(-)-2-Amino-5-phosphonopentanoic acid) and bicuculline from Tocris, Na_3_VO_4_ from New England Biolabs (Ipswich, MA, USA), glutamate from Sigma and latrunculin A from Molecular Probes, respectively.

### Plasmids and RNA interference

Various SPIN90 and Src constructs were previously described [18]. GFP- and Myc-cortactin and Myc-IRSp53 were kindly provided by Dr. Okabe (Tokyo Medical and Dental University, Japan) and Dr. Eunjoon Kim, respectively. Cofilin constructs were provided from Dr. Jun-Lin Guan (University of Michigan, Ann Arbor, Michigan) and Iryna M. Ethell (University of California, Riverside, CA). STEP constructs were a gift from Dr. Paul J. Lombroso (Yale University School of Medicine, New Haven, USA). STEP siRNA was previously described [19]. HEK293T cells were transfected for 72 h with STEP-specific siRNA alone or with GFP-SPIN90 using Lipofectamine 2000 (Invitrogen). STEP-specific siRNA was transfected into primary cultured neurons together with DNA using the calcium phosphate precipitation method. The pLifeAct-TagRFP construct was purchased from Ibidi. Slingshot constructs were a gift from Dr. Keith K. Murai (Montreal General Hospital, Quebec, Canada). Slingshot siRNA was designed as described previously [20]. Cortactin siRNA is described in an earlier report [21].

### Cell culture, transfection, and imaging

HEK293T and HeLa were cultured in Dulbecco’s modified Eagle’s medium supplemented with 10 % fetal bovine serum. For primary neuronal cultures, hippocampal and cortical neurons were collected from E18 to 19 rats and plated on poly-d-lysine-coated coverslips at a density of 4 × 10^5^ cells/60 mm dish. Neurons were cultured in Neurobasal medium (Invitrogen) supplemented with B27 (Invitrogen) and 2 mM GlutaMAX (Invitrogen). For biochemical experiments, we used cortical neurons because they provide enough cell lysate to conduct biochemical analyses (e.g., immunoprecipitation assays). Hippocampal neurons were used for cell imaging experiments and TritonX-100 insoluble fractionation assay. Neurons were transfected by a modified calcium phosphate precipitation method at DIV 11–12. To determine the effects of SPIN90 phosphorylation and translocation on dendritic spines and excitatory synapses, neurons were visualized at DIV 18–21. Cells were fixed with 4 % paraformaldehyde and 4 % sucrose in PBS and permeabilized with 0.25 % TritonX-100. They were then incubated with the appropriate primary antibodies and visualized using Alexa Fluor-conjugated secondary antibodies. F-actin was stained with Alexa Fluor 555-coupled phalloidin (Invitrogen) for 90 min at room temperature. Images were obtained using a FluoView FV 1000 confocal laser-scanning microscope equipped with 100 and 60 × oil-immersion objectives and capable of additional 3–4 × zoom.

### Bimolecular fluorescence complementation (BiFC) assay

To visualize protein interactions, BiFC assays were performed; these are based on the premise that two nonfluorescent fragments of a fluorescent protein can form a fluorescent complex, and that this association between fragments can be facilitated [22]. SPIN90 and its mutants (SPIN90 Y85/161/227E and SPIN90 Y85/161/227F) were subcloned into pBiFC-VC155, and cofilin was subcloned into pBiFC-VN173. After transfection of hippocampal neurons with these BiFC constructs, the cells were fixed and imaged using an FV1000 confocal microscope.

### Co-immunoprecipitation and Western-blot analysis

Cells were washed briefly with cold PBS and extracted for 1 h at 4 °C in modified radioactive immunoprecipitation assay buffer (50 mM Tris–HCl, pH 7.4, 150 mM NaCl, 1 % Nonidet P-40 (NP-40), 0.25 % sodium deoxycholate, 10 mM NaF, and 1 mM Na_3_VO_4_) supplemented with protease inhibitors. The extracts were then clarified by centrifugation for 10 min at 13,000 rpm, and the protein concentrations in the supernatants were determined using Bradford assays (Bio-Rad, Hercules, CA, USA). The resultant extracts were incubated with primary antibodies overnight at 4 °C. This was followed by an additional 4-h incubation with protein A/G Sepharose beads (GE Healthcare, Waukesha, WI, USA). The resulting immunoprecipitates were washed extensively with extraction buffer, separated on SDS-PAGE and transferred onto PVDF membranes. The membranes were then blocked with 5 % BSA in buffer containing 10 mM Tris–HCl, pH 7.5, 100 mM NaCl and 0.1 % Tween 20. Once blocked, the membranes were probed with primary antibodies followed by horseradish peroxidase-conjugated antibody, and the blots were detected using enhanced chemiluminescence reagent (Dogen).

### Phosphatase assay

To assay phosphatase activity in vitro, HEK293T cells transfected with GFP-SPIN90 WT and Src CA were immunoprecipitated with anti-GFP antibody and Protein A-Sepharose beads. The Protein A beads obtained after precipitation were washed first with HNTG buffer (20 mM HEPES, pH 7.5, 150 mM NaCl, 0.1 % TritonX-100 and 10 % glycerol) and then with a phosphatase reaction buffer (25 mM HEPES, pH 7.4, 5 mM EDTA and 10 mM DTT). Thereafter, the resulting bead was incubated with the amount of GST-fusion proteins containing 20 μl of phosphatase reaction buffer at 25 °C in a vibrating incubator for 15 min. The reaction was terminated by the addition of SDS sample buffer and boiling, after which the proteins were resolved by SDS-PAGE, and phosphotyrosine levels were analyzed by immunoblotting with anti-phosphotyrosine antibody.

### F- and G-actin fractionation

F/G-actin fractionation assays were performed using a kit (Cytoskeleton). Cells were washed twice with PBS and then lysed in F-actin stabilizing (LAS) buffer (50 mM PIPES, pH 6.8, 50 mM NaCl, 5 mM MgCl_2_, 5 mM EGTA (ethylene glycol tetraacetic acid), 5 % glycerol, 0.1 % NP-40, 0.1 % TritonX-100, 0.1 % Tween 20, 0.1 % β-mercaptoethanol, protease inhibitor cocktail and 1 mM ATP) by trituration with a 26.5 G syringe. The lysates were spun at 45,000 rpm for 2 h at 37 °C, after which the supernatants (G-actin) were collected and placed on ice. The pellets (F-actin) were resuspended in a volume of DW equal to that of the supernatant and containing 2 μM cytochalasin D (Sigma). The suspensions were then incubated on ice for 1 h to depolymerize F-actin. Equal amounts of G- and F-actin were subjected to immunoblotting assays using anti-actin antibody. The intensities of the bands were quantified using ImageJ software.

### Crude synaptosomal fractionation

Subcellular fractions of mouse brains were prepared as described [23, 24]. In brief, mouse whole brains were homogenized in buffered sucrose containing 0.32 M sucrose, 4 mM HEPES, pH 7.3, 1 mM MgCl_2_, 0.5 mM CaCl_2_, 10 mM NaF and 1 mM Na_3_VO_4_ supplemented with protease inhibitors. All centrifugation procedures were conducted at 4 °C. The homogenate was centrifuged at 1,000 × *g* for 10 min (yielding pellet: P1). The resulting supernatant (S1) was centrifuged at 12,000 × *g* for 15 min (yielding supernatant: S2). The resulting pellet was resuspended in buffered sucrose and centrifuged at 13,000 × *g* for 15 min (yielding pellet: P2; crude synaptosomes). For immunoprecipitation assays, the P2 fraction was extracted in modified RIPA buffer.

### Preparation of a TritonX-100 insoluble fraction and immunoblot analysis

Preparation of the TritonX-100 insoluble fraction was as described previously [7]. In brief, primary cultured neurons were extracted with TritonX-100 buffer containing 0.5 % TritonX-100, 10 mM PIPES, pH 6.8, 50 mM NaCl, 3 mM MgCl_2_ and 300 mM sucrose for 10 min at 4 °C. After extraction, the cells were washed with PBS, and the TritonX-100-insoluble fraction was collected in SDS sample buffer (50 mM Tris–HCl, pH 6.8, 2 % SDS, 2 % β-mercaptoethanol, and 10 % glycerol). Aliquots of sample solution were then subjected to SDS-PAGE and Western-blot analysis.

### Image analysis and quantification

The statistical significance of difference between means was assessed using unpaired Student’s *t* tests. In the figures with histograms, error bars indicate ± SEM. To evaluate translocation of proteins from the spines to the dendritic shafts, the spine and shaft fluorescence intensities were analyzed as the ratio of the average fluorescence intensities in the spine and the adjacent dendritic shaft. SPIN90 intensity in the spines was determined using PSD95- or Vamp2-positive puncta. SPIN90 intensity in the dendritic shafts was determined as the SPIN90 intensity in the shaft corresponding to the spine. The measurements were analyzed using MetaMorph imaging software (Universal Imaging Corporation, Bedford Hills, NY, USA). Cells were co-transfected with RFP-actin to visualize the morphology of the dendritic spines in detail. To determine spine size, about 1,000 spines (from 10 to 20 neurons) were measured under each condition. The spine heads were measured by taking the maximal width of the spine head perpendicular to the axis along the spine neck. Spine length was measured as the distance from the base of the neck to the furthest point on the spine head. For each condition, individual spine dimensions were grouped and then averaged per neuron. Spine heads and length were presented as box-and-whisker plots. The top of each box indicates the 75th percentile, the middle line indicates the median, the bottom indicates the 25th percentile, and the whiskers indicate the extent of the 10 and 90th percentiles, respectively.

## Results

### Glutamate induces redistribution of SPIN90 from spines to the dendritic shaft

**Fig. 1:**
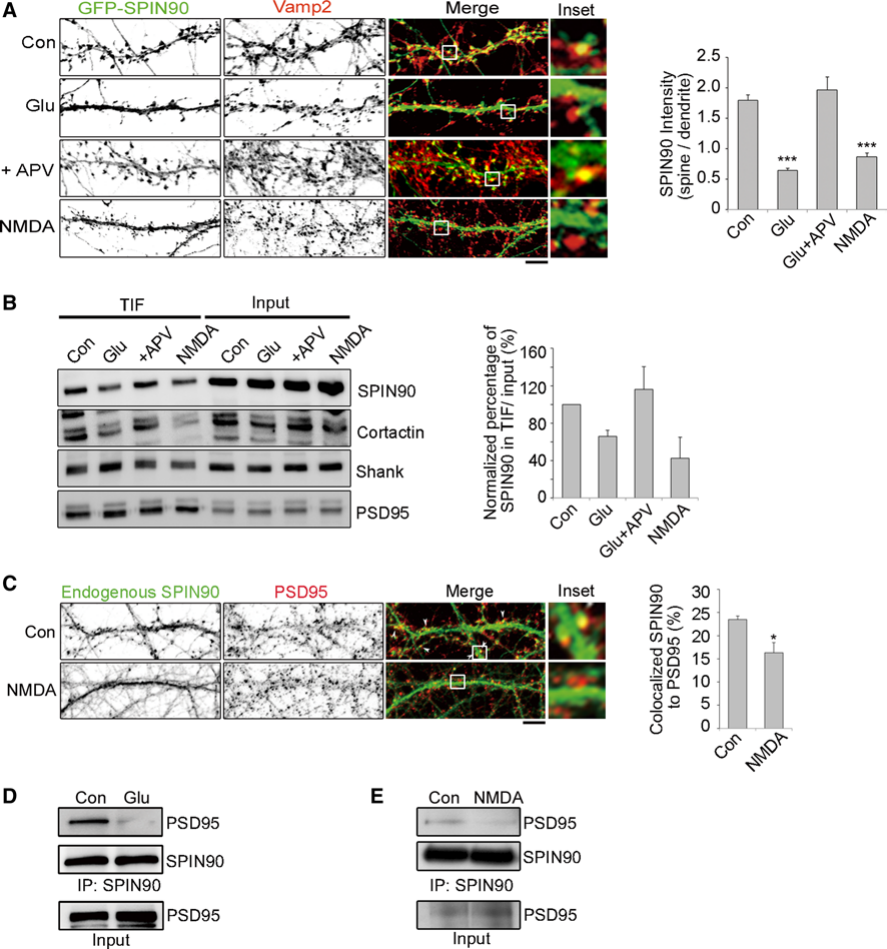
SPIN90 translocates from the spines to the dendritic shaft in response to NMDA stimulation. **a** Rat hippocampal neurons transfected with GFP-SPIN90 were treated with glutamate (glu, 100 μM for 15 min), glutamate (glu) + APV (500 μM) or NMDA (50 μM for 15 min), and then labeled with anti-Vamp2 antibody (*red*). Fluorescence intensities of GFP–SPIN90 (*green*) in the spines and in the dendritic shaft were quantified, as described in "Materials and methods". Histograms show the ratio of SPIN90 intensity in spine versus dendrite. Data represent mean ± SEM (*n* = 13–39; ***p < 0.001). *Scale bars* 5 μm. **b** Rat hippocampal neurons (DIV 19) were treated with glutamate, glutamate + APV, or NMDA, and then extracted with TritonX-100 to examine SPIN90 distribution. The ratio of SPIN90 in TIF (TritonX-100 insoluble fraction) to the input was measured and then normalized to the control. **c** Untransfected neurons were treated with NMDA at DIV 19–21 and then labeled with the indicated antibodies. Colocalization of SPIN90 (*green*) to PSD95 (*red*) was quantified. Data represent mean ± SEM (*n* = 16–18; *p < 0.05). *Scale bars* 5 μm. **d**, **e** Rat cortical neurons (DIV 19–21) stimulated with glutamate (**d**) or NMDA (**e**) were immunoprecipitated with anti-SPIN90 antibody and immunoblotted with the antibodies indicated

Little is known about the function of SPIN90 during synaptic activation, though it is known that SPIN90 localizes within dendritic spines and interacts with PSD proteins [17]. To determine whether synaptic activity regulates the localization of SPIN90 in dendritic spines, we expressed GFP-SPIN90 in cultured hippocampal neurons. Under normal growth conditions, GFP-SPIN90 was enriched in the dendritic spines, but glutamate or NMDA stimulation led to a redistribution of GFP-SPIN90 to the dendritic shaft within 15 min. Moreover, this glutamate-induced SPIN90 translocation was effectively inhibited by APV, an NMDAR antagonist (Fig. 1a). In addition, the TritonX-100 insoluble fraction prepared from cultured hippocampal neurons, which reflects the contents of the dendritic spines, exhibited a marked reduction in SPIN90 after glutamate or NMDA stimulation, and this effect was blocked when APV was present during glutamate treatment (100 % for control; 65.9 ± 6.5 % for glutamate; 116.0 ± 24.6 % for glutamate + APV; 42.5 ± 22.3 % for NMDA, Fig. 1b). Glutamate or NMDA stimulation also led to a considerable reduction in the level of cortactin in TritonX-100 insoluble fraction, but not PSD95 and Shank as previously reported [9].

To exclude an effect of SPIN90 overexpression in spines, we tested the translocation of endogenous SPIN90 in neurons. NMDA treatment significantly reduced the level of endogenous SPIN90 in the spines (Fig. 1c). As SPIN90 interacts with PSD95, a stable postsynaptic density marker [16], we tested the interaction of SPIN90 and PSD95. As we expected, SPIN90 dissociated from PSD95 by glutamate or NMDA treatment (Fig. 1d, e). Taken together, these results indicate that SPIN90 translocates from spines to the dendritic shaft upon NMDAR stimulation.

### STEP61 mediates SPIN90 dephosphorylation and translocation

NMDAR activation leads to the redistribution of many actin-binding proteins in dendritic spines, including cortactin, drebrin A, and AKAP79, as a result of actin depolymerization [9, 10, 25]. Cortactin and drebrin A are F-actin binding proteins, and latrunculin A, an actin-depolymerizing drug, induces their translocation from the spines to the shaft. We therefore tested whether actin depolymerization is the driving force behind SPIN90 translocation. Neurons treated with latrunculin A or NMDA exhibited the pronounced actin depolymerization and the disappearance of GFP-cortactin and endogenous drebrin A from dendritic spines, as previously reported [9, 10] (Supplementary Fig. 1a, b). However, GFP-SPIN90 remained in the spines even after latrunculin A treatment, indicating that actin depolymerization alone is not sufficient for driving SPIN90 translocation observed in NMDA treatment (Supplementary Fig. 1c). Hence, we examined whether the phosphorylation status of SPIN90 contributes to SPIN90 translocation.

**Fig. 2:**
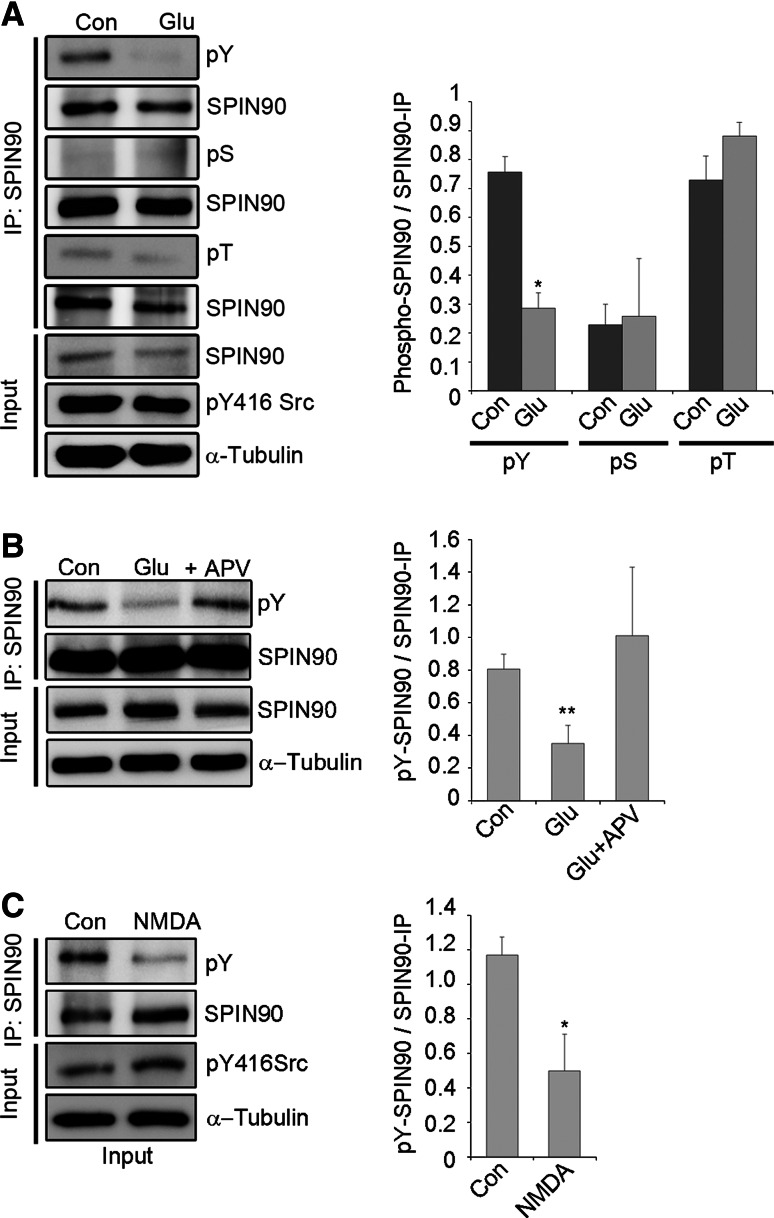
NMDA induces SPIN90 tyrosine-dephosphorylation. **a–c** SPIN90 phosphorylation was examined in rat cortical neurons (DIV 19–21) treated with glutamate (100 μM for 15 min) (**a**), glutamate + APV (500 μM) (**b**), or NMDA (**c**). Each of the lysates was subject to immunoprecipitation and Western blotting with the indicated antibodies. The ratio of phosphorylated SPIN90 to immunoprecipitated SPIN90 was measured and then presented as a histogram. Data represent mean ± SEM (*p < 0.05, **p < 0.01)

First, we addressed the status of SPIN90 phosphorylation under synaptic activation. Interestingly, tyrosine phosphorylation, but not threonine or serine phosphorylation, of SPIN90 was dramatically reduced after glutamate or NMDA stimulation (Fig. 2a, c), and this reduction was inhibited by the NMDAR antagonist, APV (Fig. 2b), providing a possible link between SPIN90 phosphorylation and its NMDA-induced translocation. Next, we used 55 mM KCl and 50 μM bicuculline, instead of NMDA, to replicate the physiological conditions for NMDA receptor activation effectively [9, 26, 27]. In our experiments, stimulation with each compound induced SPIN90 translocation from the spine to shaft, as well as dephosphorylation (Supplementary Fig. 2).

Since we previously reported that SPIN90 is tyrosine-phosphorylated by a Src kinase in dendritic spines [18], we assumed that NMDA-induced SPIN90 dephosphorylation is due to the reduction of Src kinase activity. However, Src activity was unchanged upon NMDA treatment, as indicated by the level of pY416-Src (Supplementary Fig. 3a) [28]. Therefore, the reduction in SPIN90 tyrosine-phosphorylation under NMDA treatment is unlikely due to a reduction in Src kinase activity.

We next focused on the possible involvement of a protein tyrosine phosphatase in SPIN90 dephosphorylation. As expected, SPIN90 translocation and dephosphorylation was significantly inhibited by sodium orthovanadate (NaV), a nonspecific tyrosine phosphatase inhibitor (Supplementary Fig. 3b, c), suggesting that NMDA stimulation activates tyrosine phosphatase, which is responsible for SPIN90 dephosphorylation. We previously identified Src phosphorylation sites (Y85, Y161, Y227) in SPIN90 and found that the phosphomimetic mutant (SPIN90 YE; Y85/161/227E) were enriched in dendritic spines [18]. In addition, following NMDA stimulation, dendritic spines of neurons expressing GFP-SPIN90 wild-type (WT) displayed a significant loss of fluorescence. By contrast, NMDA had no effect on the fluorescence in spines of neurons expressing GFP-SPIN90 YE, indicating that translocation of the phosphomimetic mutant was effectively suppressed (Supplementary Fig. 3c) and tyrosine-dephosphorylation in SPIN90 is critical for its translocation to the dendritic shafts.

**Fig. 3:**
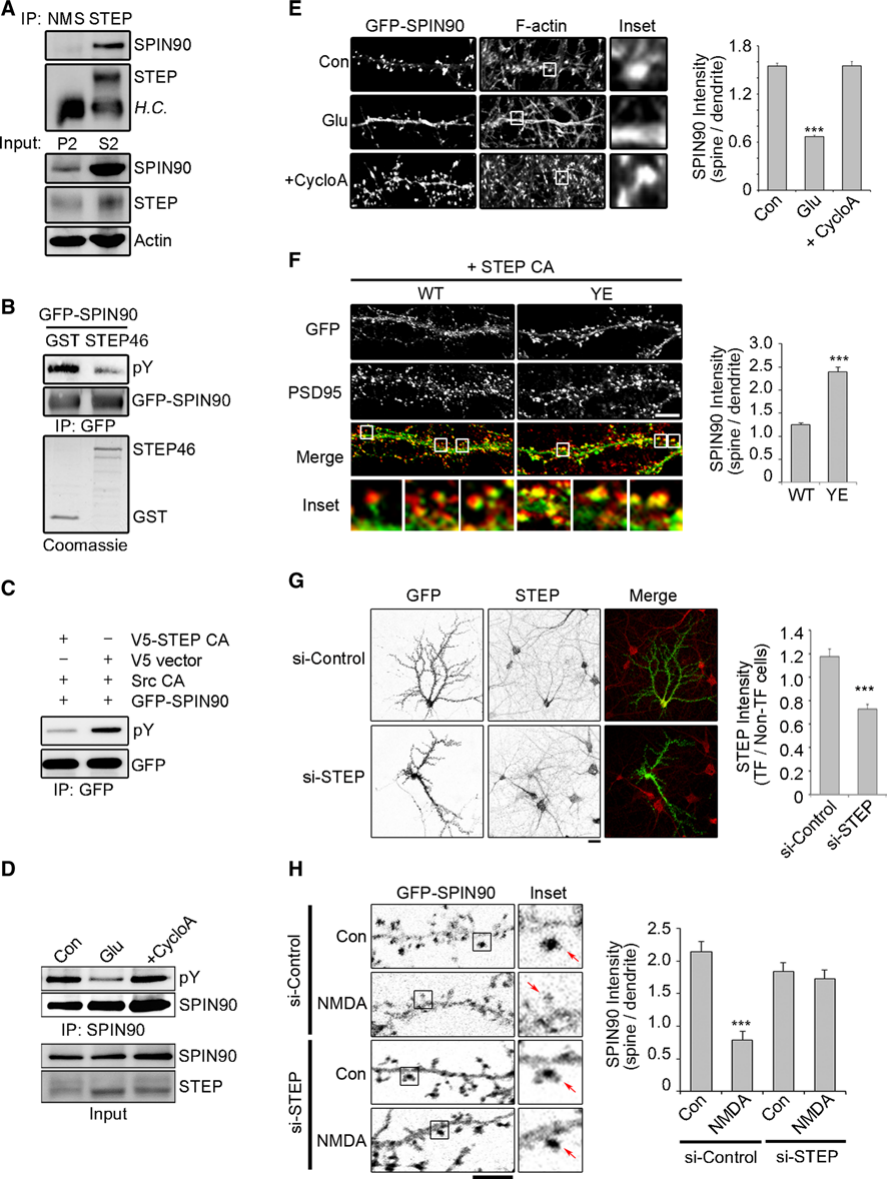
STEP61 catalyzes SPIN90 dephosphorylation and promotes SPIN90 translocation. **a** Mouse brain lysates were fractionated into crude synaptosomal (P2) and cytosolic fractions (S2), and the extracted P2 fractions were immunoprecipitated with anti-STEP antibody or normal mouse serum (NMS). The fractionation of P2 and S2 is shown on the bottom panel.* H.C.* indicates heavy chains. **b** In *vitro* STEP-mediated phosphatase assay. HEK293T cells cotransfected with GFP-SPIN90 and Src CA were immunoprecipitated with anti-GFP antibody. The immunoprecipitated complex was subject to in *vitro* phosphatase assay with GST-STEP46. **c** SPIN90 phosphorylation was examined in HEK293T cells co-transfected with Src CA plus V5-STEP CA or control vector (V5). **d** Pre-incubation of cortical neurons (DIV 19–21) with cyclosporine A (5 μM for 10 min) inhibits glutamate-induced SPIN90 dephosphorylation. **e** Hippocampal neurons expressing GFP-SPIN90 WT were pre-incubated with cyclosporin A (5 μM for 10 min) before glutamate treatment (100 μM for 15 min) (*n* = 7–15, ***p < 0.001). *Scale bar*, 5 μm. **f** Cultured hippocampal neurons were transfected with GFP-SPIN90 WT or YE plus STEP61 CA, and the localization of GFP-SPIN90 (*green*) was analyzed (*n* = 13–17; ***p < 0.001). *Scale bar*, 5 μm. **g** To test the efficiency of siRNA against STEP61, STEP siRNA with GFP vector were transfected into hippocampal neurons, and followed by immunostaining with anti-STEP antibody (*red*). STEP intensity was measured and presented as histograms (*n* = 12; ***p < 0.001). *Scale bar*, 20 μm. **h** Knockdown effects of STEP siRNA on SPIN90 translocation in hippocampal neurons. The ratio of SPIN90 intensity in spine versus dendrite was measured. *Arrows* indicate GFP-SPIN90 in the spines. Data represent mean ± SEM (*n* = 5–11; ***p < 0.001). *Scale bar*, 5 μm

To search for a potential tyrosine phosphatase for SPIN90 dephosphorylation, we first tested the interaction of SPIN90 and STEP61 because STEP61 is a tyrosine phosphatase which is activated by NMDA stimulation [29]. Immunoprecipitation assays using a crude synaptosomal fraction from mouse brain showed that SPIN90 and STEP61 were readily co-immunoprecipitated (Fig. 3a). In vitro phosphatase assays using STEP46, which contains the conserved phosphatase domain of STEP61, demonstrated that the phosphorylated GFP-SPIN90 by Src CA (constitutively active form of Src; Src Y527F) in HEK293T cells was tyrosine-dephosphorylated by GST-STEP46 but not by GST alone (Fig. 3b). In addition, overexpression of STEP CA (constitutively active form of STEP; STEP S221A) rendered SPIN90 dephosphorylated (Fig. 3c). Therefore, we examined STEP inhibitor blocks SPIN90 translocation and dephosphorylation. Because there is no direct STEP inhibitor found, we used cyclosporine A, which inhibits calcineurin, a STEP activator. We found that the translocation and dephosphorylation of SPIN90 induced by glutamate was suppressed in cells treated with cyclosporine A (Fig. 3d, e). To examine the possibility that Slingshot, a downstream molecule of calcineurin and activator of cofilin, regulates SPIN90 translocation, we tested the effects of Slingshot on SPIN90 dephosphorylation in HEK293T cells. The SPIN90 phosphorylation level was unaffected by Slingshot WT overexpression whereas phospho-cofilin was decreased, as reported previously [30] (Supplementary Fig. 4a). In addition, upon knockdown of Slingshot with specific siRNA, SPIN90 translocation was triggered in the presence of NMDA (Supplementary Fig. 4b, c). However, F-actin staining was detected in the spines of Slingshot siRNA-transfected neurons, suggesting blockage of NMDA-induced actin depolymerization. Therefore, we conclude that inhibition of NMDA-induced SPIN90 translocation by cyclosporine A is attributable to suppression of STEP, but not Slingshot. In parallel, most of SPIN90 WT was located at the dendritic shaft when STEP61 CA was co-expressed, but SPIN90 YE remained in the spines, indicating that SPIN90 dephosphorylation and translocation are primarily dependent on STEP61 activity (Fig. 3f). To confirm that dephosphorylation of SPIN90 by STEP is essential for SPIN90 translocation, STEP was knocked down using STEP-specific siRNAs and the knockdown of STEP was verified by immunocytochemical assays in cultured hippocampal neurons (Fig. 3g). In the STEP-knockdown cells, NMDA-evoked translocation of SPIN90 to the dendritic shafts was dramatically reduced, whereas in control cells, most of SPIN90 was translocated to the dendritic shaft (Fig. 3h). Collectively, these data indicate that STEP61 is essential for NMDA-induced SPIN90 translocation.

### Phosphorylated SPIN90 interacts with cofilin

**Fig. 4:**
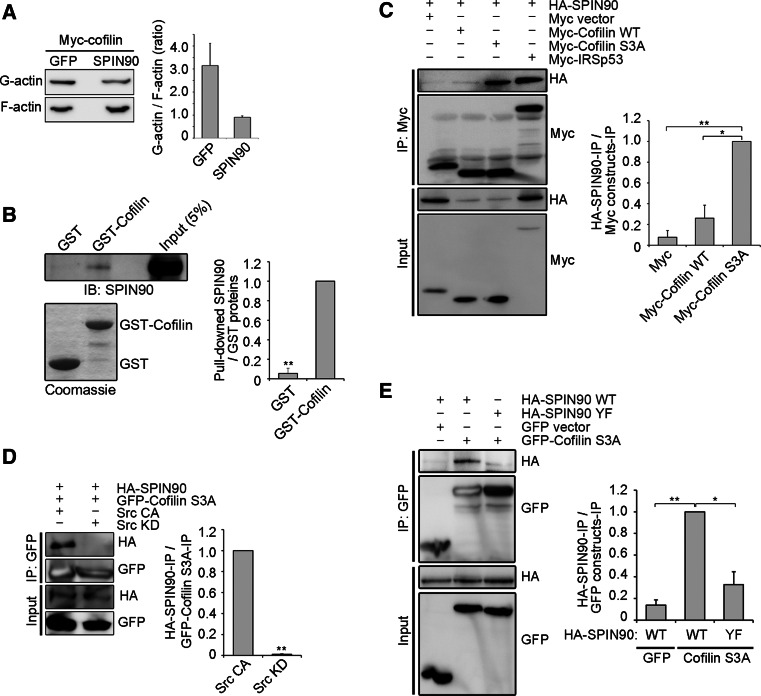
SPIN90 binding to active cofilin is dependent on its phosphorylation. **a** HEK293T cells were co-transfected with Myc-cofilin plus GFP-SPIN90 or GFP. Cellular G- and F-actin were fractionated by ultracentrifugation as described in "Materials and methods". **b** Cell extracts from cortical neurons (DIV20) were incubated with GST or GST-cofilin protein. **c** HEK293T cells were co-transfected with HA-SPIN90 WT plus Myc-cofilin WT or S3A and subject to immunoprecipitation. IRSp53 serves as a positive control for a SPIN90 binding protein. **d** The binding of cofilin S3A to HA-SPIN90 is enhanced in HEK293T cells expressing Src CA compared to Src KD. **e** SPIN90 phospho-deficient mutant (SPIN90 YF) displays the decreased interaction with cofilin compared to SPIN90 WT

Our previous findings that neurons expressing SPIN90 phospho-deficient mutant (SPIN90 YF; Y85/161/227F) exhibited remarkable spine shrinkage compared to SPIN90 WT or phosphomimetic mutant transfected neurons [18] led us to test whether SPIN90 dephosphorylation is involved in actin depolymerization responsible for spine shrinkage [31]. We first performed in vivo G-actin/F-actin fractionation assays. G-actin/F-actin ratio was lower in HEK293T cells co-transfected with Myc-cofilin and GFP-SPIN90 than in cells expressing Myc-cofilin and GFP, reflecting inhibition of cofilin-mediated actin depolymerization by SPIN90 (3.15 ± 0.90, *n* = 3 for GFP; 0.96 ± 0.06, *n* = 3 for GFP-SPIN90; Fig. 4a).

To identify interaction between SPIN90 and cofilin, we performed GST pull-down assay with cortical neuron extracts and GST-cofilin or GST. GST-cofilin readily pulled-down SPIN90 from cortical neuron extracts but GST did not (Fig. 4b). In immunoprecipitation assays using transfected HEK293T cells, SPIN90 WT interacted with cofilin S3A, a constitutively active form of cofilin, but not with cofilin WT (Fig. 4c). As shown in Supplementary Fig. 5, most of the overexpressed myc-cofilin WT was present in the phosphorylated (inactive) form. In addition, cofilin S3A readily interacted with SPIN90 WT when co-expressed with Src CA, but not when co-expressed with Src KD, a kinase-dead form of Src (Fig. 4d). This confirmed that SPIN90 phospho-deficient mutant (YF) did not interact with cofilin S3A (Fig. 4e).

**Fig. 5:**
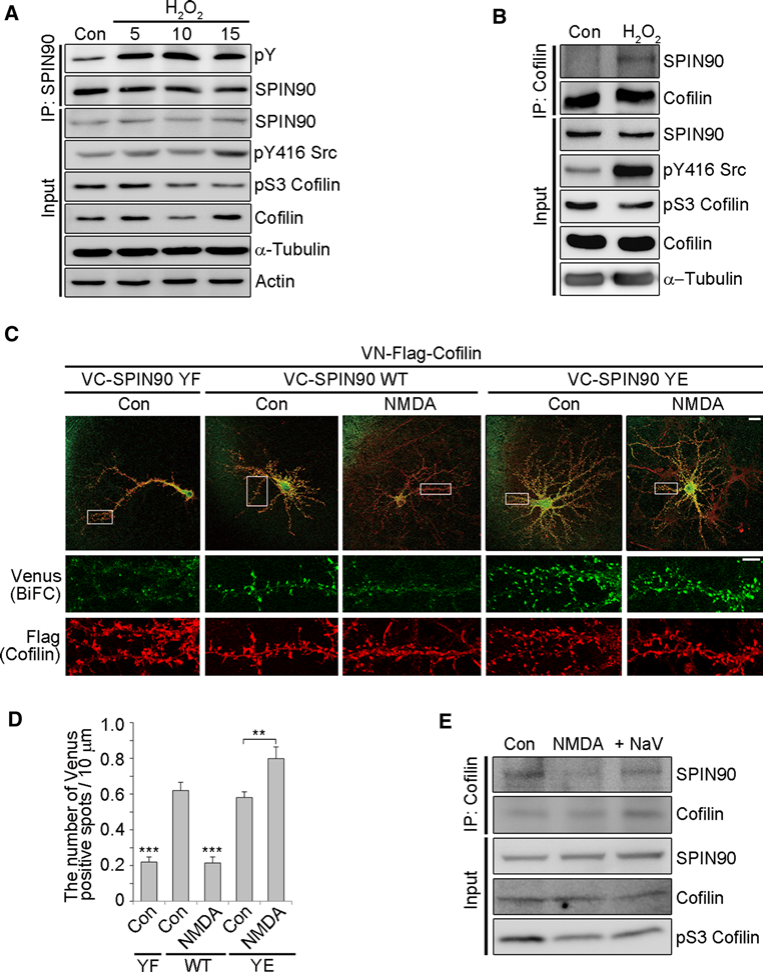
SPIN90 interacts with cofilin in neurons through a phosphorylation-dependent mechanism. **a** Lysates from cortical neurons (DIV 19) treated with H_2_O_2_ (0.5 mM for indicated time) were immunoprecipitated using anti-SPIN90 antibody and immunoblotted with the antibodies indicated on the *right*. **b** Lysates from cortical neurons (DIV 19) treated with H_2_O_2_ (0.5 mM for 15 min) were immunoprecipitated using anti-cofilin antibody and immunoblotted with the indicated antibodies. **c**, **d** In BiFC assays, hippocampal neurons co-expressing VN-flag-cofilin plus VC-SPIN90 constructs were treated with 50 μM NMDA for 15 min and monitored for BiFC (*Venus*) signal. VN-cofilin immunostained with anti-Flag antibody (*red*) is used as an internal control. The number of *venus* spots was counted and represented as mean ± SEM (*n* = 8–20; ***p < 0.001, **p < 0.01). *Scale bar* for low magnification, 20 μm; *scale bar* for high magnification, 5 μm. **e** Sodium orthovanadate (NaV, 1 mM for 15 min) was applied onto cortical neurons, prior to NMDA application

To examine the interaction between phosphorylated SPIN90 and active cofilin (dephosphorylated cofilin) in vivo, we carried out co-immunoprecipitation assays in cortical neurons treated with H_2_O_2_. As the previous reports [32, 33], H_2_O_2_ treatment effectively induced Src phosphorylation (active form) and cofilin dephosphorylation (active form) in cortical neurons (Fig. 5a). Phosphorylated SPIN90 was also detected in H_2_O_2_-treated neurons (Fig. 5a) and its interaction with active cofilin (dephosphorylated cofilin) was further confirmed (Fig. 5b).

Next, SPIN90-cofilin interaction was tested using BiFC assays, which measure venus (green) signals emitted when two molecules fused to VN (Venus N-terminus) or VC (Venus C-terminus) interact with each other. When neurons were co-transfected with VN-fused Cofilin and VC-fused SPIN90, BiFC signals were prominent in spines of neurons co-expressing SPIN90 YE and cofilin, but not in neurons co-expressing SPIN90 YF and cofilin (Fig. 5c, d). NMDA stimulation significantly reduced BiFC signals in spines of neurons co-expressing SPIN90 WT and cofilin, whereas it slightly increased BiFC signals in neurons co-expressing SPIN90 YE and cofilin, probably due to the increase in active cofilin level by NMDA stimulation. Moreover, pre-incubation of cortical neurons with sodium orthovanadate (NaV), which blocks SPIN90 dephosphorylation (Supplementary Fig. 3b), significantly increased the interaction of SPIN90 with cofilin upon even NMDA treatment (Fig. 5e). In neurons treated with sodium orthovanadate, dephosphorylated cofilin was maintained (Supplementary Fig. 6; Fig. 5e), in turn, leading to significantly increased interactions with SPIN90, even upon NMDA treatment (Fig. 5e). Taken together, these findings suggest that phosphorylated SPIN90 is able to bind the active form (dephosphorylated) of cofilin, thereby probably sequestering active cofilin from F-actin in spines.

### Phosphorylated SPIN90 inhibits cofilin-mediated actin depolymerization

**Fig. 6:**
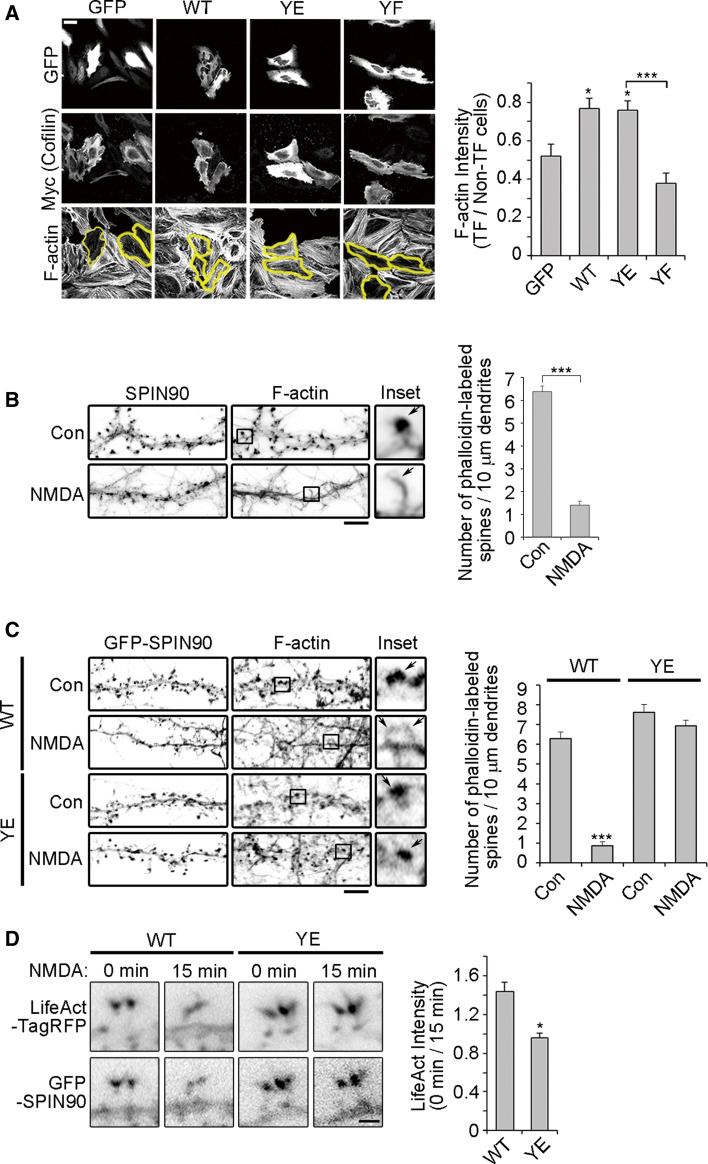
Phosphorylated SPIN90 inhibits cofilin-mediated actin depolymerization. **a** HeLa cells were co-transfected with Myc-cofilin plus GFP-SPIN90 WT, YE, YF or GFP, as indicated. Cells were stained with anti-Myc antibody or phalloidin to visualize F-actin. The transfected cells are marked with *yellow lines*. The ratio of the F-actin intensity in the transfected and non-transfected cells is presented as histograms (*n* = 13–58; *p < 0.05, ***p < 0.001). *Scale bars*, 10 μm. **b**, **c** Untransfected neurons and those expressing GFP-SPIN90 WT or YE were stimulated with 50 μM NMDA for 15 min and stained with SPIN90 (**b**) and phalloidin (**b**, **c**). The *arrows* indicate the location of spines. The number of phalloidin-labeled spines was counted. Data are presented as mean ± SEM (**b**, *n* = 10–11; **c**, *n* = 9–41; ***p < 0.001). *Scale bars*, 5 μm. **d** Hippocampal neurons were co-transfected with GFP-SPIN90 WT or YE plus LifeAct-TagRFP. The images were taken before (0 min) and after NMDA stimulation (15 min). LifeAct-TagRFP relative intensity in spine versus shaft was measured for each spine. The ratio of relative intensity at 0–15 min was calculated and presented as histograms (*n* = 3–4; *p < 0.05). *Scale bars*, 1 μm

To test whether phosphorylated SPIN90 inhibits cofilin-mediated actin depolymerization, cells were co-transfected with SPIN90 and cofilin and F-actin staining was examined. HeLa cells expressing GFP-SPIN90 YE or WT exhibited prominent F-actin staining, even in the presence of overexpressed Myc-cofilin. By contrast, cofilin-mediated actin depolymerization markedly occurred in HeLa cells cotransfected with GFP-SPIN90 YF and cofilin or GFP alone, indicating that phosphorylated SPIN90 participates in the regulation of cofilin activity (Fig. 6a).

It is well known that cofilin in neurons is activated through its dephosphorylation within several minutes of NMDA treatment and NMDA-induced loss of F-actin from spines is mediated by cofilin [34, 35]. Therefore, we tested whether the loss of F-actin from spines by NMDA treatment is related to SPIN90 dephosphorylation and translocation. NMDA treatment induced a significant reduction in the phalloidin-stained F-actin level in the spines of untransfected (Fig. 6b) and GFP-SPIN90 WT-expressing neurons (Fig. 6c). By contrast, F-actin staining was still prominent in the spines of neurons expressing GFP-SPIN90 YE even after NMDA stimulation (Fig. 6c). In live cell imaging, NMDA dramatically reduced the LifeAct-TagRFP signal, which visualizes F-actin in living cells [36], from the spines of GFP-SPIN90 WT neurons, and this loss of signal was accompanied by the redistribution of GFP-SPIN90 WT to the dendritic shafts (Fig. 6d). The same NMDA treatment had no effect on the LifeAct-TagRFP signal in spines expressing SPIN90 YE, indicating that actin depolymerization was effectively suppressed (Fig. 6d). These results suggest that SPIN90 dephosphorylation and translocation is a prerequisite for cofilin activation in dendritic spines.

### Phosphorylated SPIN90 blocks spine head shrinkage and alters synaptic activity

**Fig. 7:**
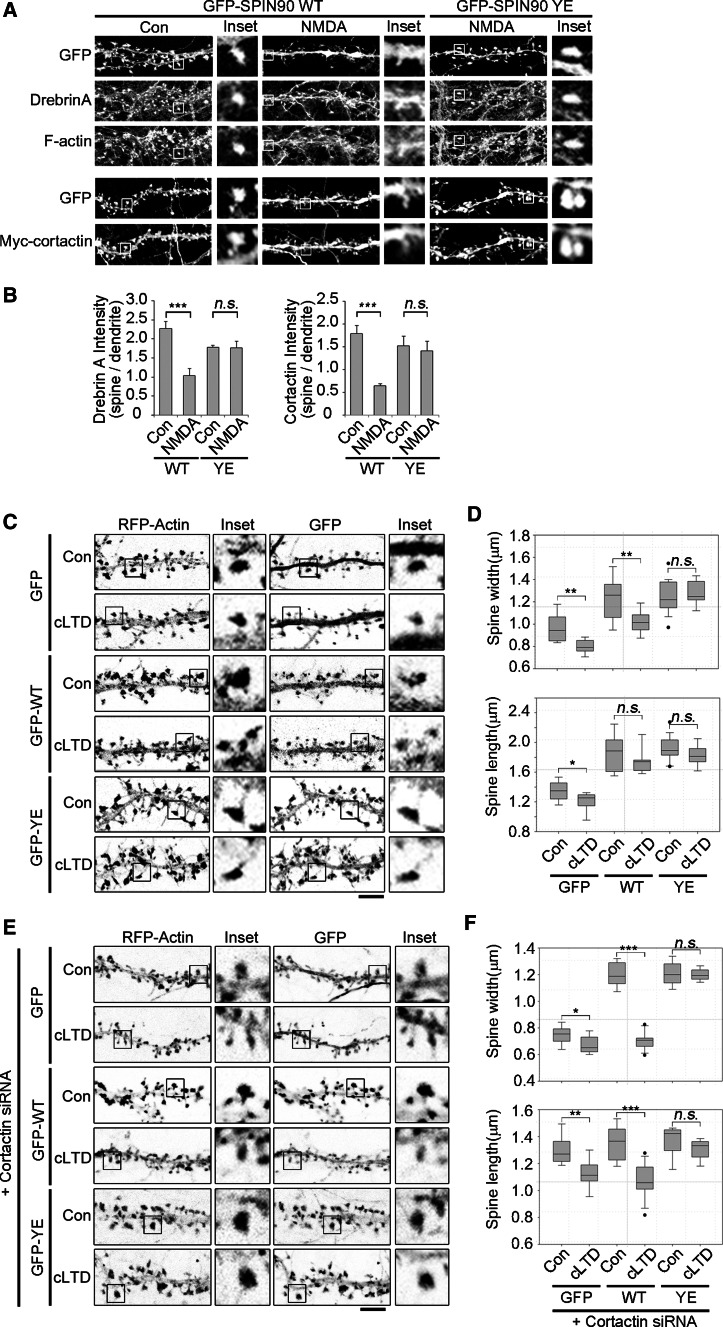
SPIN90 phosphorylation suppresses NMDA-induced spine shrinkage. **a** Hippocampal neurons transfected with GFP-SPIN90 WT or YE at DIV 10–12 were subject to immunofluorescence assays at DIV 19–21. NMDA-induced translocation of drebrin A and Myc-cortactin from the spines to the dendritic shaft were decreased in neurons expressing SPIN90 YE compared to SPIN90 WT. *Scale bars*, 5 μm. **b** The translocation of cortactin and drebrin A was analyzed as in “Materials and methods” (*n* = 7–20; ***p < 0.001). **c** Analysis of spine morphology in hippocampal neurons overexpressing SPIN90 WT or YE upon chemical LTD (cLTD) induction. Hippocampal neurons were co-transfected with RFP-actin plus GFP-SPIN90 WT or YE at DIV 10–12, and then treated with NMDA (20 μM for 3 min), followed by additional incubation in growth medium for 40 min (cLTD induction) at DIV 19–21. **d** Spine head width and length before and after cLTD induction were analyzed using box-and-whisker plots (*n* = 10–20; *p < 0.05, **p < 0.01). *n.s.* non-significant. *Error bars*, SEM. *Scale bars*, 5 μm. **e** Analysis of spine morphology in hippocampal neurons overexpressing GFP, SPIN90 WT, or YE in cortactin knockdown neurons. Hippocampal neurons were co-transfected with RFP-actin plus GFP-SPIN90 constructs and cortactin siRNA at DIV 10–12, and cLTD induced at DIV 19–21. **f** Spine heads, widths, and lengths before and after cLTD induction analysis using box-and-whisker plots (*n* = 9–12; *p < 0.05, **p < 0.01, ***p < 0.001). *n.s.* non-significant. *Error bars*, SEM. *Scale bars*, 5 μm

Cortactin and drebrin A, two F-actin binding proteins, are known to be translocated from dendritic spines to the shaft on actin depolymerization in response to NMDAR activation [9, 10]. Our finding that SPIN90 phosphorylation blocks NMDA-induced actin depolymerization through cofilin inhibition prompted us to investigate whether redistribution of cortactin and drebrin A is also regulated by SPIN90 phosphorylation. Immunocytochemical assays showed that overexpression of SPIN90 YE significantly inhibited translocation of drebrin A and Myc-cortactin, so that they remained in the spines, even after NMDA treatment (Fig. 7a, b).

Because cofilin is crucial for spine shrinkage by chemical LTD induction (cLTD) [4], we examined whether SPIN90 phosphorylation inhibits spine shrinkage after cLTD induction. As predicted, we found that SPIN90 phosphorylation blocked spine head shrinkage. The spine head width was reduced in neurons expressing SPIN90 WT after cLTD induction, but was unchanged in neurons expressing SPIN90 YE (Fig. 7c, d). As cortactin and drebrin are important factors for maintenance of F-actin in spines, it is necessary to determine whether these proteins affect the inhibition of cLTD-induced spine shrinkage by SPIN90 YE. Initial examination of whether cortactin and drebrin bind SPIN90 revealed no interactions. In contrast, PSD95 bound to SPIN90, as shown previously (Supplementary Fig. 7a). Next, we used specific siRNA to block the effects of cortactin in SPIN90 YE-transfected spines (Supplementary Fig. 7b). GFP-SPIN90 YE expression led to ready blockage of cLTD-induced spine shrinkage, even in the presence of cortactin siRNA (Fig. 7e, f). Taken together, our data indicate that SPIN90 phosphorylation/dephosphorylation contributes to the regulation of dendritic spine head shrinkage mediated by cofilin.

## Discussion

Actin is highly enriched in dendritic spines, where it anchors to many scaffolding proteins and serves as a key determinant of spine morphology, thereby modulating synaptic function [37]. In particular, brief NMDA receptor activation induces the breakdown of F-actin, such as actin depolymerization, which is known to be mediated by calcineurin signaling. When calcium enters the spines, calcineurin is activated, which triggers cofilin activation, leading to actin depolymerization [34, 35, 38]. However, it remained unclear how NMDA-induced actin reorganization is achieved. In this paper, we have demonstrated that SPIN90 dephosphorylation is a prerequisite for NMDA-mediated actin depolymerization in dendritic spines. We showed that NMDA treatment induces tyrosine-dephosphorylation of SPIN90 by activating STEP61, which promotes translocation of SPIN90 from the dendritic spine to the shaft. However, as long as SPIN90 remains in the phosphorylated state, it binds to cofilin, thereby blocking actin depolymerization. Therefore, SPIN90 dephosphorylation is crucial for cofilin-mediated actin depolymerization in NMDA-treated hippocampal neurons.

Synaptic plasticity is modulated by protein phosphorylation and dephosphorylation. One of the key phosphatases in NMDAR-mediated synaptic depression is STEP, a tyrosine phosphatase, which is activated on NMDAR stimulation [29]. It has been reported that STEP suppresses LTP induction, while potentiating LTD, implying that STEP is involved in the regulation of synaptic strength and memory formation [39–41]. In this study, we found that NMDA induces tyrosine-dephosphorylation of SPIN90 by STEP, resulting in SPIN90 withdrawal from the postsynaptic compartment. Recently, we demonstrated that SPIN90 phosphorylation is crucial for its directed targeting to postsynaptic sites, which also brings about enhanced synaptic activity [18]. In addition, phosphorylation of SPIN90 enhances interactions with scaffolding proteins (PSD95 and Shank) in the postsynaptic compartment. This readily expands the structural capacity of postsynaptic densities, resulting in increased spine head width and synaptic activity, which is apparent in SPIN90 YE (phosphomimetic form)-expressing neurons [18]. Moreover, fluorescent imaging and electrophysiological data [18] showed that SPIN90 YF (phospho-deficient form)-transfected neurons display reduced spine size and synaptic activity compared to SPIN90 WT or YE (phosphomimetic form)-transfected neurons. Thus, there is a correlation between the synaptic function of STEP and the properties of dephosphorylated SPIN90. Collectively, these findings suggest that SPIN90 dephosphorylation by STEP is a critical step in synaptic depression mediated by NMDAR activation.

Several studies have demonstrated that cofilin-binding proteins are involved in the regulation of synaptic function by modulating cofilin activity. A prominent example is LIMK, a ubiquitously expressed actin-binding kinase [42]. Cofilin phosphorylation in hippocampal neurons was severely impaired in LIMK1/2 double-knockout mice, resulting in abnormal spines with thicker necks [43]. In contrast, β-arrestin-2 transports cofilin towards the spine on LTD induction, thereby enhancing spine shrinkage [4]. This suggests that cofilin activity is important for spine morphogenesis, depending on its phosphorylation. Our present results indicate that SPIN90 acts in additional ways to modulate cofilin activity in dendritic spines. That actin depolymerization in response to NMDAR activation was completely blocked in neurons expressing SPIN90 YE, prompted us to hypothesize that SPIN90 might bind to cofilin, thus hindering its actin-depolymerizing activity. Consistent with that idea, phosphorylated SPIN90 bound to cofilin and inhibited cofilin activity. The BiFC assays further confirmed that phosphorylated SPIN90 binds to active cofilin in spines. Thus, we propose that SPIN90 binding to active cofilin, thereby blocking cofilin to bind to F-actin, is dependent on its phosphorylation/dephosphorylation status, which might be regulated by STEP on synaptic stimulation.

SPIN90 participates in many actin-related functions, such as lamellipodia, growth cone, dendritic spine formation and endocytosis [13, 14, 16, 17, 44–46]. It is well known that SPIN90 regulates actin polymerization by activating N-WASP and Arp2/3 complex [44, 46]. However, in this study, we showed that SPIN90 also regulates actin depolymerization, but negatively, by binding to active cofilin, thereby modulating cofilin activity. Thus, it is the first study to prove that SPIN90 regulates actin depolymerization.

In response to NMDAR activation, SPIN90 is dephosphorylated and translocated from the spines to the dendritic shaft. If this translocation is blocked so that SPIN90 remains in the spines, despite synaptic activity, we can postulate several possible outcomes. First, the inhibition of SPIN90 dephosphorylation could enable its interaction with Shank and PSD95 to be sustained within the spines. Synaptic clustering of phosphorylated SPIN90 with Shank and PSD95 increases both the size and density of dendritic spines [16–18]. It is well known that Shank promotes spine maturation and enlargement [47], and that PSD95 is involved in increasing spine density and the number of synapses [48]. In addition, activity-dependent alterations in spine shape are regulated by PSD95 through the regulation of trafficking of PSD proteins [49]. Thus, interaction of SPIN90 with Shank and PSD95, independently of NMDAR activation, would inhibit reorganization of the PSD in response to synaptic activity. Secondly, the presence of the phosphomimetic mutant SPIN90 YE in spines blocked activity-dependent actin depolymerization. The inhibition of actin depolymerization by jasplakinolide in hippocampal slices inhibits spine shrinkage and synaptic depression after LFS stimulation [50]. The loss of spines due to shrinkage reduces the size of paired presynaptic boutons, which in turn leads to synaptic losses [51], suggesting that actin depolymerization is closely associated with spine shrinkage and, ultimately, synaptic loss. Therefore, the inhibition of redistribution of SPIN90 in spines could lead to the possible malfunction of neural networks, resulting in neurological diseases. Thirdly, because actin depolymerization was inhibited in neurons expressing SPIN90 YE, despite NMDA stimulation, the redistribution of the F-actin binding proteins cortactin and drebrin A was also inhibited. Cortactin binds directly to the Arp2/3 complex and promotes nucleation of actin filaments [52], and the binding of drebrin A to profilin promotes actin polymerization in spines [11]. That both of these proteins promote the formation of F-actin suggests that their presence in spines would stabilize F-actin, which could explain the lack of spine shrinkage in SPIN90 YE-expressing neurons. However, it is interesting to note that SPIN90 YE inhibited cLTD-induced spine shrinkage in absence of cortactin (Fig. 7e, f). Collectively, these scenarios are consistent with the notion that SPIN90 translocation and phosphorylation/dephosphorylation are key determinants of spine morphology and synaptic plasticity.

In this study, we have defined a role for SPIN90 phosphorylation and dephosphorylation which governs actin depolymerization and dendritic spine morphology by regulating cofilin activity. We suggest that SPIN90 may function as a key modulator in initiating actin reorganization and spine shrinkage.

### Electronic supplementary material

Below is the link to the electronic supplementary material.

Supplementary material 1 (DOC 5192 kb)
