# Supplementary material for: SPIN90 dephosphorylation is required for cofilin-mediated actin depolymerization in NMDA-stimulated hippocampal neurons
Source: Cell Mol Life Sci. 2013 Jun 14;70(22):4369–83. doi: 10.1007/s00018-013-1391-4 (PMC3825632; doi:10.1007/s00018-013-1391-4)
Supplement: Supplementary file 1 — Supplementary material 1 (DOC 5192 kb) [file 18_2013_1391_MOESM1_ESM.doc]

**Supplementary Materials**

**SPIN90 Dephosphorylation is required for Cofilin-mediated Actin Depolymerization in NMDA-stimulated Hippocampal Neurons**

In Ha Cho^1^, Min Jung Lee^1^, Dae Hwan Kim^1^, Bora Kim^1^, Jeomil Bae^1^, Kyu Yeong Choi^1^,

Seon-Myung Kim^2^, Yun Hyun Huh^1^, Kun Ho Lee^3^, Chong-Hyun Kim^4^, and Woo Keun Song^1**^¶

**Affiliations:**

^1^Bio Imaging and Cell Dynamics Center, School of Life Sciences, Gwangju Institute of Science and Technology, Gwangju, 500-712, Korea.

^2^Program of Developmental Neurobiology, Institute of Molecular Medicine and Genetics, Department of Neurology, Medical College of Georgia, Augusta, GA 30912, USA.

^3^Department of Marine Biology, Chosun University, Gwangju 501-759, Korea

^4^Center for Neural Science, Korea Institute of Science and Technology, Seoul and Department of Neuroscience, University of Science and Technology, Daejeon, Korea.

**Inventory of Supplementary Information**

**Supplementary Figures**:

Fig. S1: NMDA-induced actin depolymerization is insufficient for SPIN90 translocation.

**Fig. S2.** Treatment with KCl or bicuculline induces SPIN90 translocation and dephosphorylation.

Fig. S3: Inhibition of tyrosine phosphatase suppresses NMDA-induced SPIN90 translocation.

**Fig. S4.** The phosphorylation level of SPIN90 is unaffected by Slingshot.

**Fig. S5.** Overexpressed cofilin WT represents the inactive form.

**Fig. S6.** Cofilin dephosphorylation is unaffected by sodium orthovanadate in NMDA-stimulated neurons.

**Fig. S7.** Cortactin and drebrin do not bind SPIN90.

**
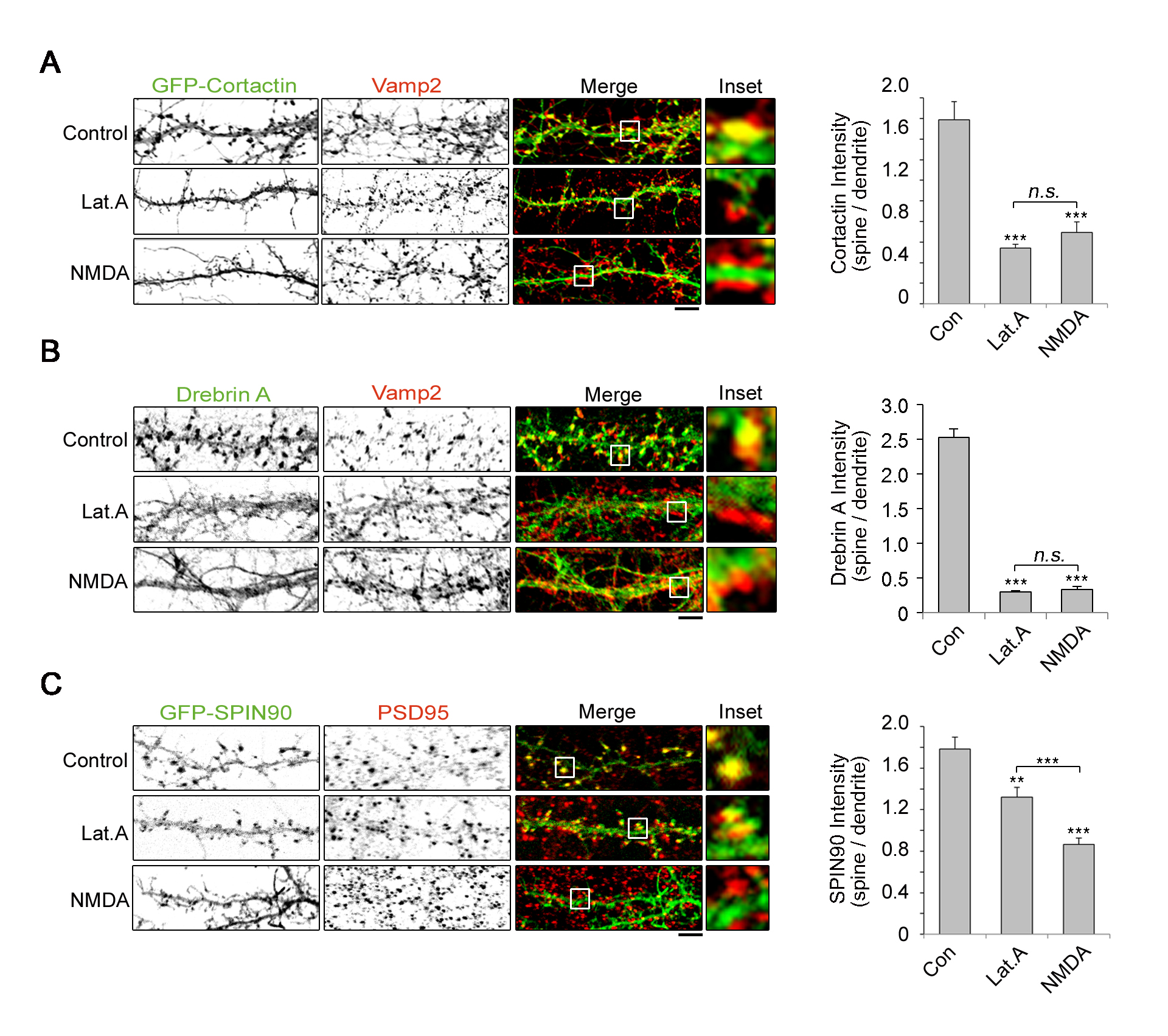
**

**Fig. S1.** NMDA-induced actin depolymerization is insufficient for SPIN90 translocation. (**A, B**) GFP-cortactin (**A**) and endogenous drebrin A (**B**) were redistributed by NMDA (50 μM for 15 min) or latrunculin A (5 μM for 15 min) treatments. Fluorescence intensities of GFP-cortactin or drebrin A in the spines and in the dendritic shaft were quantified, as described in the **Materials and Methods** section. The histograms show the ratio of intensity in spine to dendrite. Data represent means ± SEM (n=9-36; ***P < 0.001). *n.s.*, non-significant. Scale bars, 5 μm. (**C**) GFP-SPIN90 transfected neurons was treated with NMDA or latrunculin A, and then labeled with anti-PSD95 antibody (n=19-84; **P < 0.01, ***P < 0.001).


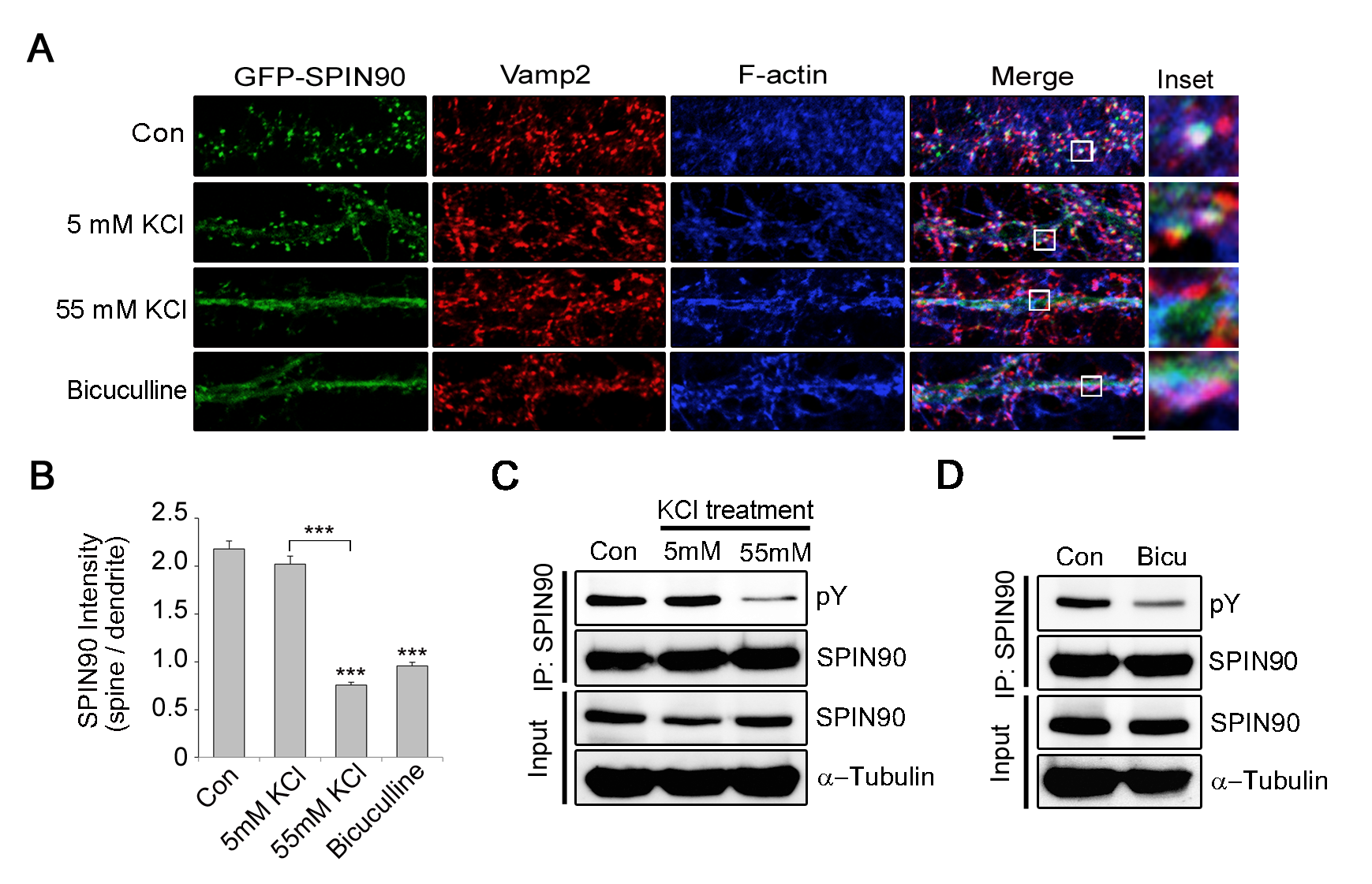


**Fig. S2.** Treatment with KCl or bicuculline induces SPIN90 translocation and dephosphorylation. (**A**) Rat hippocampal neurons transfected with GFP-SPIN90 were treated with KCl (5 mM and 55 mM each for 3 min) or bicuculline (50 μM for 1 h), and labeled with anti-Vamp2 antibody (red) and phalloidin (blue). Fluorescence intensities of GFP-SPIN90 (green) in the spines and dendritic shaft were quantified, as described in Materials and Methods. Histograms show the ratio of SPIN90 intensity in spines vs. dendrites. Data are presented as means ± SEM (n=11-15; *** P < 0.001). Scale bars, 5 μm. (**B**) SPIN90 phosphorylation was examined in rat cortical neurons (DIV 19-21) treated with KCl (5 mM and 55 mM each for 3 min) and bicuculline (50 μM for 1 h). Lysates were subjected to immunoprecipitation and Western blotting with the indicated antibodies.

**
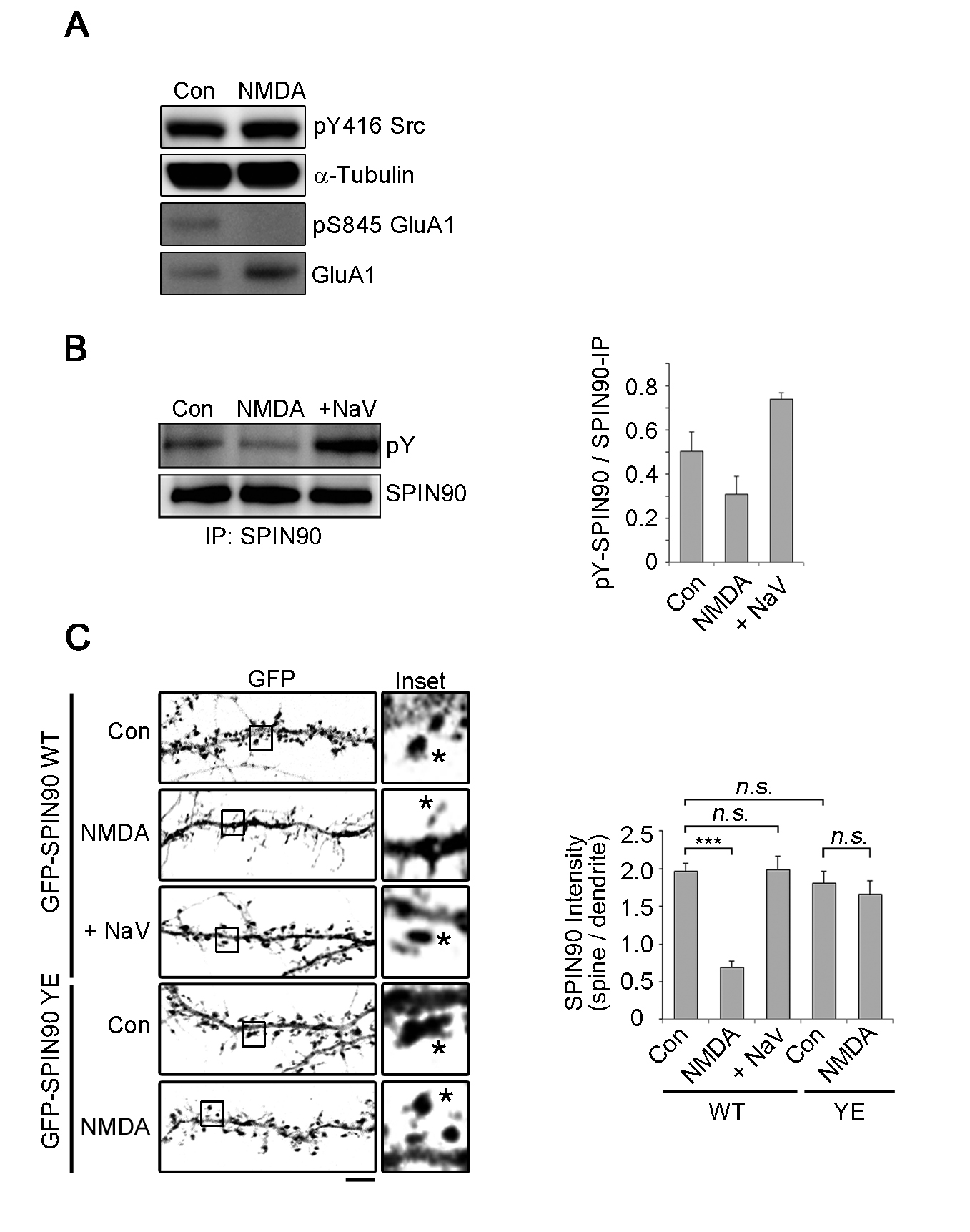
**

**Fig. S3.** Inhibition of tyrosine phosphatase suppresses NMDA-induced SPIN90 translocation. (**A**) NMDA stimulation has no effect on Src activity even though NMDA worked properly, as proved by the reduced phosphorylation of GluA1. (**B**) Pre-incubating cortical neurons with NaV (1 mM for 15 min) inhibits NMDA‑induced SPIN90 dephosphorylation. (**C**) Neurons expressing GFP-SPIN90 WT were pre-incubated with NaV (1 mM for 15 min) before NMDA treatment. GFP-SPIN90 YE-transfected neurons were stimulated with NMDA. Asterisks indicate spines. Histograms represent means ± SEM (for WT, n=6-7, ***P < 0.001; for YE, n=7-12, P = 0.572). Scale bars, 5 μm.


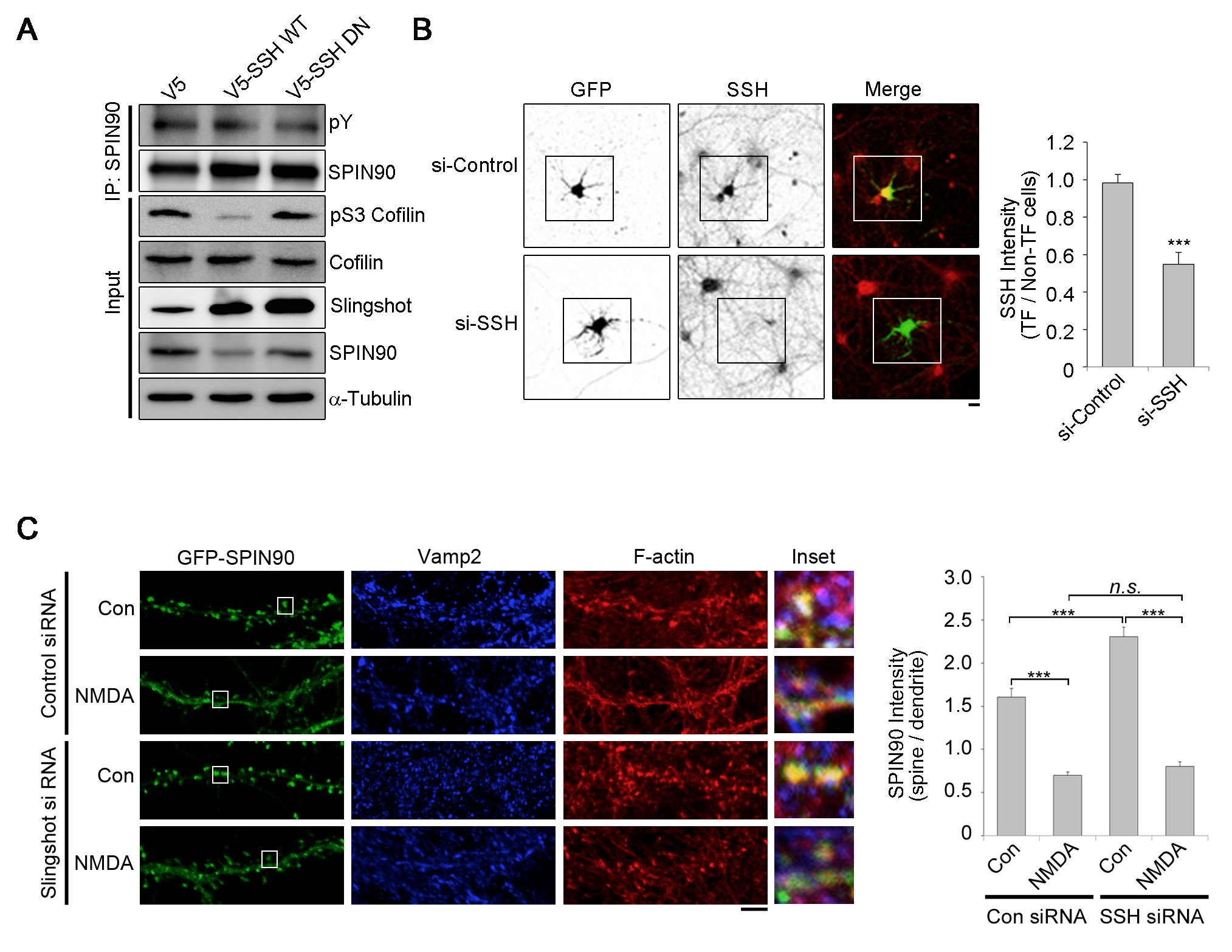


**Fig. S4.** The phosphorylation level of SPIN90 is unaffected by Slingshot. (**A**) SPIN90 phosphorylation was examined in HEK293T cells transfected with V5-Slingshot WT (V5-SSH) or V5-Slingshot DN (V5-SSH DN, dominant-negative; phosphatase-inactive mutant; C393S) or control vector (V5). Cell lysates were immunoprecipitated with anti-SPIN90 antibody and immunoblotted with the indicated antibodies. The pS3-cofilin level served as a positive control for Slingshot activity. (**B**) To test the efficiency of Slingshot siRNA, hippocampal neurons were transfected with Slingshot siRNA plus GFP vector and immunostained with anti-Slingshot antibody (red). Slingshot intensity was measured and presented as histograms (n=9-14; ***P < 0.001). Scale bar, 10 μm. (**C**) Knockdown effects of Slingshot siRNA on SPIN90 translocation in hippocampal neurons. The ratio of SPIN90 intensity in spines vs. dendrites was measured. Data are presented as means ± SEM (n=9-14; ***P < 0.001). *n.s.*, non-significant. Scale bar, 5 μm.

**
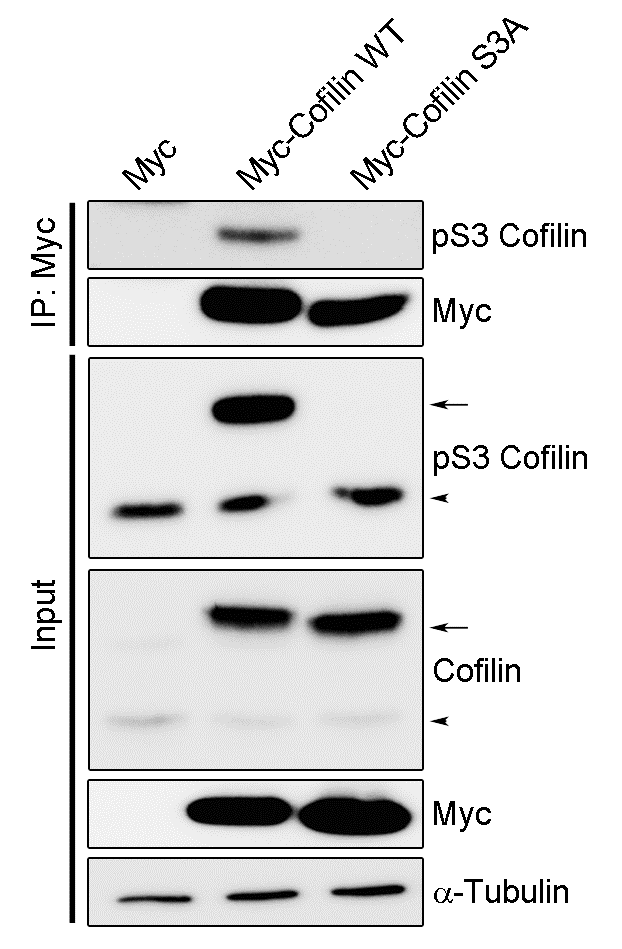
**

**Fig. S5.** Overexpressed cofilin WT represents the inactive form of cofilin. HEK293T cells were transfected with Myc-cofilin WT, Myc-cofilin S3A or Myc empty vector, and subjected to immunoprecipitation. Myc-cofilin WT displayed increased pS3-cofilin levels, compared to cofilin S3A mutant. The arrow indicates overexpressed Myc-cofilin and arrowheads signify endogenous cofilin.

**
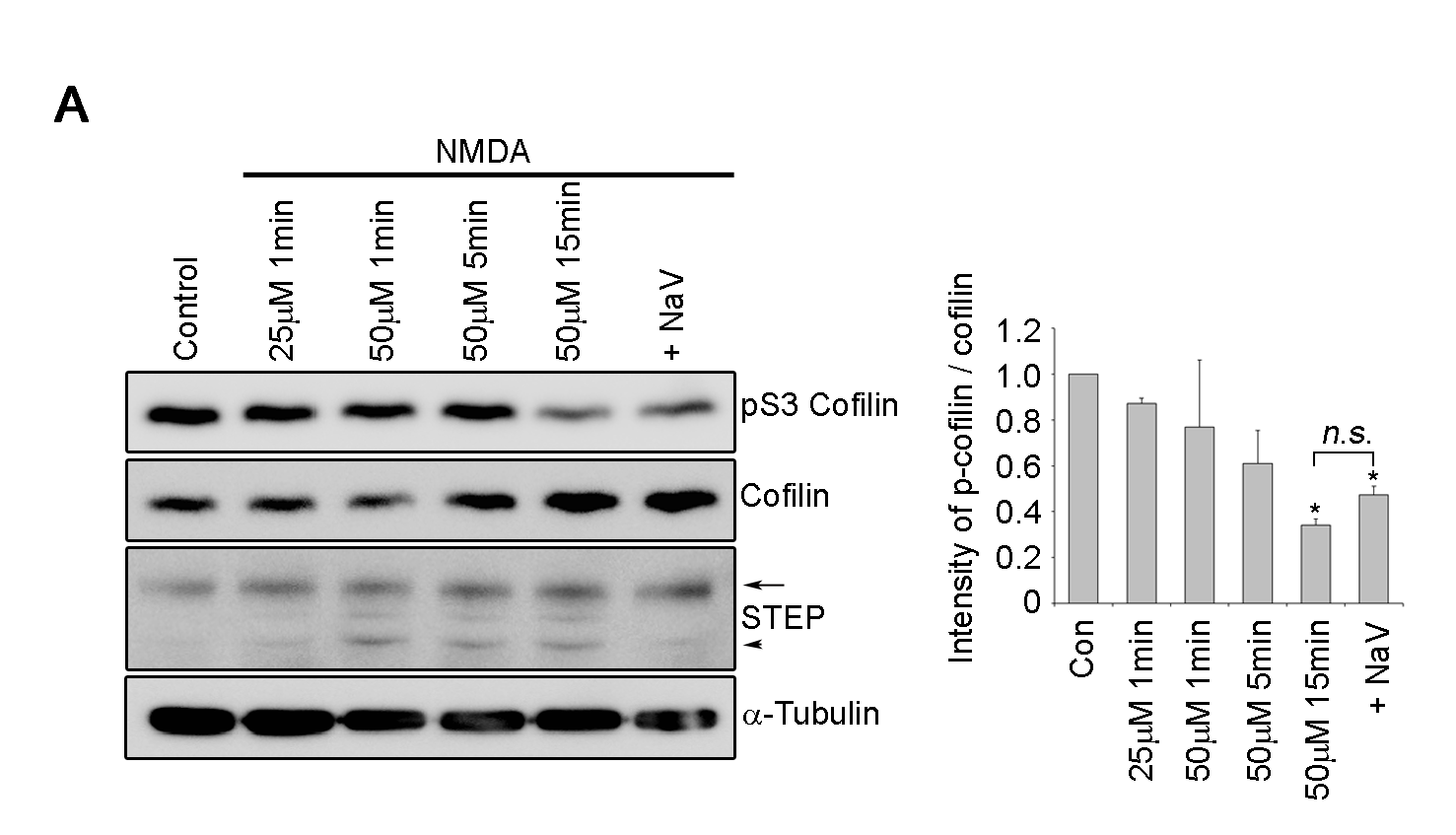
**

**Fig. S6.** Cofilin dephosphorylation is unaffected by sodium orthovanadate in NMDA-stimulated neurons. Rat hippocampal neurons (DIV 19-21) were treated with NMDA alone or together with NaV (1 mM for 15 min), and subjected to immunoblotting. The arrow indicates phosphorylated STEP and arrowheads signify dephosphorylated STEP. The ratio of phosphorylated to total cofilin was measured and presented as a histogram. Data represent means ± SEM (* P < 0.05). *n.s.*, non-significant.

**
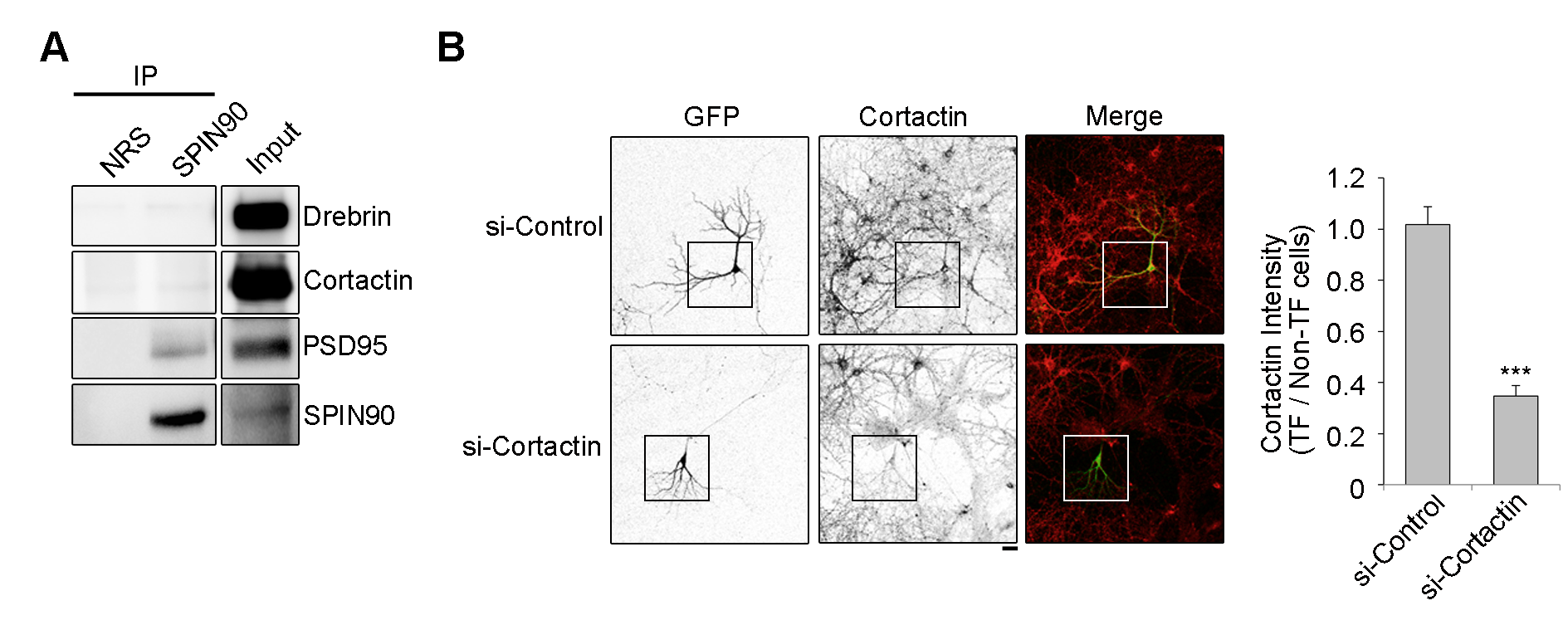
**

**Fig. S7.** Cortactin and drebrin do not bind SPIN90. (**A**) Rat cortical neurons (DIV19-21) were immunoprecipitated with anti-SPIN90 antibody or normal rabbit serum, and immunoblotted with the indicated antibodies. (**B**) To test the efficiency of cortactin depletion, hippocampal neurons were transfected with cortactin siRNA in GFP vector and immunostained with anti-cortactin antibody (red). Cortactin intensity is presented as a histogram (n=10-11; ***P < 0.001). Scale bar, 20 μm.
